# IL-13 mediates collagen deposition via STAT6 and microRNA-135b: a role for epigenetics

**DOI:** 10.1038/srep25066

**Published:** 2016-04-26

**Authors:** Steven O’Reilly, Marzena Ciechomska, Nicola Fullard, Stefan Przyborski, Jacob M. van Laar

**Affiliations:** 1 Faculty of Health and Life Sciences, Northumbria University, Newcastle Upon Tyne, NE1 8ST, United Kingdom; 2 Institute of Cellular Medicine Faculty of Medical Sciences, MRG 4^th^ Floor Cookson Building Framlington Place, Newcastle Upon Tyne, NE2 4HH; 3 National Institute of geriatrics rheumatology and rehabilitation, Warsaw, Poland; 4 Institute of Immunology and Experimental Therapy, Polish Academy of Science, Wroclaw, Poland; 5 School of Biological and Biomedical Sciences, Durham University, Durham United Kingdom; 6 Department of Rheumatology and Clinical Immunology University Medical Centre Utrecht, Utrecht, The Netherlands

## Abstract

Systemic sclerosis is an autoimmune connective tissue disease in which T cells play a prominent role. We and others have previously demonstrated a role for T cell-derived IL-13 in mediating the induction of collagen in dermal fibroblasts and that blockade with IL-13 antibodies attenuates this increase. In this study we want to probe the signalling that underpins IL-13 mediated matrix deposition. Isolated dermal fibroblasts were incubated with recombinant IL-13 and gene expression by qRT-PCR was performed for collagen1A1 and TGF-β1. Small interfering RNA (siRNA) was used to knock down STAT6 and a small molecule inhibitor was also used to block this pathway. MiR-135b was transfected into fibroblasts plus and minus IL-13 to see if this miR plays a role. miR-135b was measured in systemic sclerosis fibroblasts isolated from patients and also in serum. Results showed that IL-13 increased collagen expression and that this is independent from TGF-β1. This is dependent on STAT6 as targeting this blocked induction. MiR-135b reduces collagen induction in fibroblasts and scleroderma fibroblasts have lower constitutive levels of the miR. We further demonstrate that miR135b is repressed by methylation and may include MeCP2. In conclusion we show that STAT6 and miR-135b regulate IL-13-mediated collagen production by fibroblasts.

Systemic sclerosis (SSc) is a polygenic, idiopathic connective tissue disease characterised by autoimmunity, vascular damage, inflammation and fibrosis. Activation of quiescent fibroblasts into myofibroblasts that express alpha-smooth muscle actin and secrete excessive extracellular matrix molecules is critical to the fibrosis that underpins the disease pathogenesis^1^ and underpins fibrosis whatever organ is affected.

Tissue fibrosis leads to excessive scarring that ultimately leads to loss of organ function and currently there is no disease modifying drug approved for treatment and there is substantial morbidity and mortality. Experimental studies show a clear link between the inflammation and fibrosis and many diverse cell types are involved in the inflammation and fibrosis. It has been shown that monocytes and T cells infiltrate the dermis in SSc especially prominent in early disease. T cells are particularly prominent early in the disease. Activation of T cells has been shown by the expression of T cell activation markers^2^. SSc is characterised by elevated IL-4 and IL-13 levels in serum^3^,^4^ and abnormalities in Th2 cells. Indeed there is a correlation between IL-13 serum levels and nailfold capillaroscopy abnormalities in SSc patients^5^. We demonstrated that T cell isolated from skin have upregulated expression of Tumour Necrosis Factor-α (TNF-α) receptors and Interleukin-13 (IL-13)^6^ in SSc patients. Engagement of IL-13 (or IL-4) to its receptor IL-13R and the shared receptor IL-4Rα promotes Janus Kinase (JAK) activation that in turn leads to phosphorylation of STAT6, homodimer or heterodimer formation via their amino terminal domains, and translocation to the nucleus where they bind DNA, influencing gene expression in many cell types. STAT6 itself is important for the polarisation of naïve T cells to Th2 effector cells^7^. This activation of STAT6 leads to activation of the transcription factor GATA3 which regulates the expression of Th2 cytokines such as IL-4 and IL-13 thus differentiating the T cells to a Th2 phenotype^8^ which appears to be the dominate T cell phenotype in SSc^3^,^6^.

IL-13 and IL-4 have been shown to augment collagen gel contraction in *in vitro* models using pulmonary fibroblasts, suggesting matrix remodelling^9^. Furthermore, overexpression of IL-13 in the lung in transgenic mice causes inflammation and lung fibrosis^10^, and an IL-13 inhibitor blocks the development of fibrosis in a Th2 dominant animal model in which animals are exposed to shistosomiasis^11^. Disruption of the IL-4 gene in the Tight skin mouse (Tsk), a model of SSc in which the gene for fibrllin is mutated, reduces the fibrosis^12^. However, the mechanism by which IL-13 cause’s fibrosis is still to be elucidated.

MicroRNAs are small (around 21 nucleotides long) RNA molecules that function to regulate protein expression by translational inhibition or mRNA degradation through binding of the seed region with a complementary match site in the 3′UTR of the target mRNA^13^. It is now known that there are many miRs in the genome and that each miR can target hundreds of genes, thus the level of regulation of expression is huge. Emerging evidence suggest that miRs are involved in virtually all cellular processes including growth, differentiation, apoptosis and fibrosis^14^ and evidence has now been accrued that they are perturbed in multiple diseases. In SSc it has been found that there are altered expression of various miRs and one of the most important is miR-29a which regulates collagen directly through binding to its 3′UTR^15^ and enforced overexpression of miR-29a reduces collagen levels in SSc dermal fibroblasts. MiR-29a has also been identified in fibrosis of the heart after myocardial infarction^16^. Strategies that restore the levels of miR29a *in vitro* and *in vivo* reduce fibrosis by targeting its mRNA target collagen1A1^15^. Thus modulation of miRs *in vivo* is a possible therapeutic option in fibrosis. However, relatively few miRs have been described in SSc as compared to other diseases such as cancer or diabetes for example. MiRS themselves can be regulated epigenetically by methylation for instance and also histone modifications can also alter their expression. Thus all the epigenetic modifications can each affect one another and underpins the complexity of epigenetics. This study shows that miR-135b targets STAT6 and attenuates IL-13-induced collagen expression.

## Results

### IL-13 induces collagen independent of TGF-β signalling

**Figure 1:**
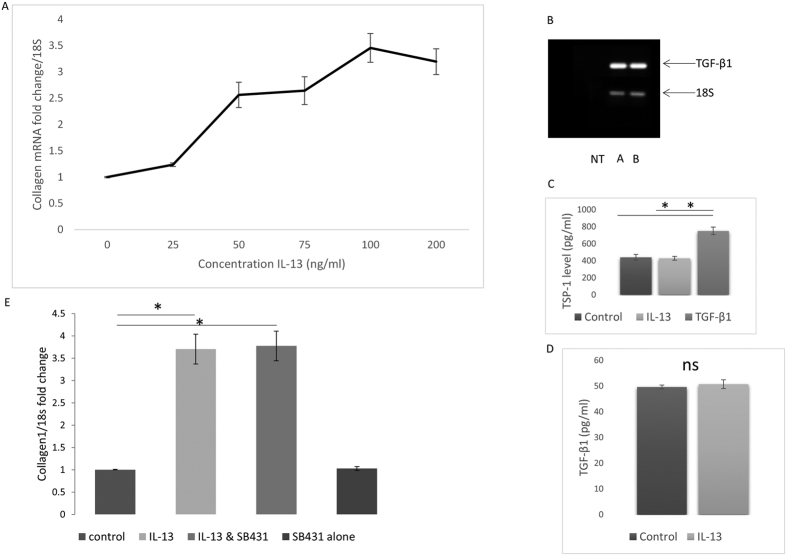
Dose response relationship of IL-13 and collagen induction. (**A**) Dermal fibroblasts were incubated with increasing doses of IL-13 *in vitro* and collagen1A1 was measured by qRT-PCR. Data shown is the fold change normalised to 18S housekeeping mRNA data is mean and standard deviation n = 4. (**B**) Representative semi-quantitative RT-PCR of TGF-β1 after stimulation of cells with IL-13 for 24 hours PCR cycling was performed for 25 cycles and amplicons were electrophoresed on a 2% agarose gel with ethidium bromide incorporated within and imaged under UV light. 18S was used as an internal control NT: No template control, A untreated, B: IL-13 (100 ng/ml) 24 hours treatment. (**C**) Fibroblast conditioned medium was analysed by ELISA for TSP-1 after removal of FCS and stimulation with IL-13 (100 ng/ml). TGF-β1 stimulation served as a positive control, data are the mean and SD from 4 independent experiments. (**D**) Fibroblast conditioned media was measured for TGF-β1 after stimulation with IL-13 (**E**) Dermal fibroblasts were treated with the TGF-βR blocker SB431542 or untreated and then stimulated with IL-13 (100 ng/ml) and after 24 hours collagen1A1 mRNA was quantified by qRT-PCR and normalised to 18S. Data is the mean and SD n = 4.

Because we had previously demonstrated that IL-13 derived from T cells induces collagen expression *in vitro* we sought to confirm this in a ‘cleaner’ system free from other T cell cytokines ‘contaminating’ the system. To confirm that IL-13 induces collagen in isolated dermal fibroblasts we performed a dose response study. Figure 1 demonstrates that 100 ng/ml of IL-13 induced the highest fold change of collagen1A1 mRNA expression peaking at 3.5 fold change in expression (normalised to 18 S). This elevation of collagen was also found on the protein levels with a 72% increase in collagen compared to control after IL-13 treatment (100 ng/ml). There was no change in the expression of the myofibroblast marker α-smooth muscle actin expression by qRT-PCR at any concentration of IL-13. It has also been suggested that IL-13 induces TGF-β1 expression and release and that TGF-β1 is responsible for the collagen increase in a transgenic animal model of IL-13 overexpression causing lung fibrosis^17^. We did not observe any significant increase in TGF-β1 expression after IL-13 stimulation by semi q-RT-PCR (Fig. 1B). Because TGF-β can also be activated from in its latent form to a biologically active motif by the matricellular protein Thrombospondin-1 (TSP-1)^18^,^19^ we measured the levels of TSP-1 in the conditioned media. ELISA of TSP-1 levels in conditioned media after stimulation of dermal fibroblasts revealed no increase in secreted levels of TSP-1 between control or IL-13 stimulation (mean value: 440 vs 428 pg/ml, *P* =>0.05 Student’s t-test). TGF-β1 stimulation served as a positive control and was significantly different compared to control and IL-13 treated (Fig. 1C). Direct measurement of TGF-β1 also showed no significant increase in expression (Fig. 1D). The target gene of TGF-β1 CTGF was also measured by RT-PCR and this was also not changed by the addition of exogenous recombinant IL-13 (data not shown). To confirm the role of TGF-β in IL-13 mediated collagen1A1 induction cells were pre treated with the TGF-βR inhibitor SB431542 and then stimulated with IL-13. Blockade of TGF-β signalling did not significantly attenuate IL-13-mediated induction of collagen (Fig. 1E), thus indicating that TGF-β signalling is independent of this effect. We further examined the expression of BMP and Activin Membrane-Bound Inhibitor (BAMBI) which is part of the TGF family and is a negative regulator of TGF-β1 signalling and found that this was not different after IL-13 (1 fold control versus 0.89 IL-13 stimulated expression).

### IL-13 utilises STAT6 in collagen induction

**Figure 2:**
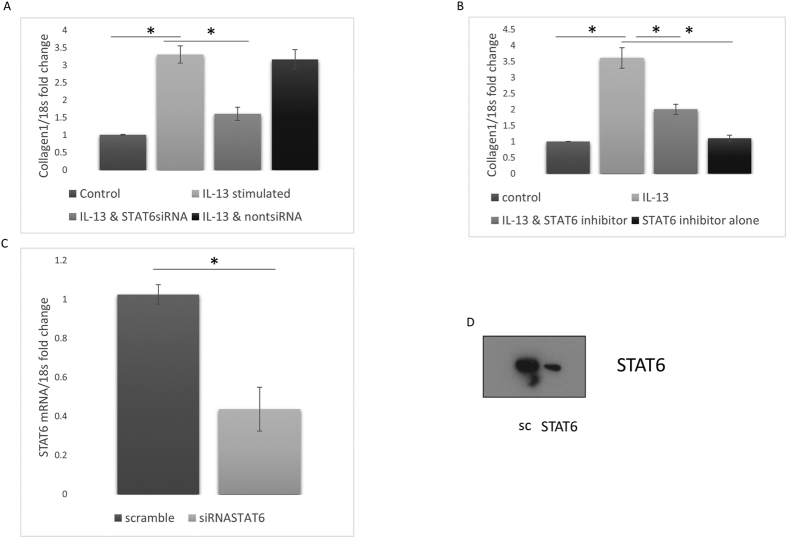
IL-13 mediates collagen production via STAT6 signalling. (**A**) Dermal fibroblasts were transfected with smartpool siRNA against STAT6 or none targeting siRNA at equal concentrations after 24 hours medium was replaced with serum free medium containing IL-13 (100 ng/ml) after a further 24 hours in culture qRT-PCR was performed for collagen1A1. (**B**) Dermal fibroblasts were incubated with IL-13 (100 ng/ml), the STAT6 inhibitor (40 nM) and IL-13 or AS1517499 alone after 24 hours qRT-PCR was performed for collagen1A1. Data is the mean and SD and is normalised to 18 S and untreated was set to 1 n = 5. (**C**) Significant reduction in STAT6 after transfection with specific STAT6 siRNA. Data is fold change compared to scrambled none specific siRNA after transfection using DharmaFECT1 transfection. Data are the mean and SD n = 4 significant difference Students t test. (**D**) Representative western blot of STAT6 after scrambled control siRNA transfection or specific STAT6 siRNA after transfection using DharmaFECT1 n = 4.

To examine the role of the transcription factor Signal Transducer and Activator of Transcription-6 (STAT-6) in IL-13 mediated collagen expression we used siRNA to deplete dermal fibroblasts of STAT6. It was demonstrated that IL-13 addition (100 ng/ml) induced collagen1A1 expression, but this was reduced by pre-treatment with specific siRNA against STAT6 significantly to 1.6 fold change compared to control. Importantly, this was not reduced by the introduction of equivalent concentration of scrambled control siRNA indicating its specificity (Fig. 2A) (P =<0.05 ANOVA). Furthermore, chemical inhibition of STAT6 with the STAT6 specific inhibitor AS1517499^20^ (40 nM) lead also to a reduction of IL-13 mediated increases in collagen expression as compared to control untreated cells (Fig. 2B). Figure 2C demonstrates the efficiency of the siRNAs against STAT6 by measuring STAT6 transcripts with a 60% reduction in STAT6 transcripts after transfection of the STAT6 siRNA and also protein expression (Student’s t test) (Fig. 2D protein reduction).

### miR-135b regulates IL-13-mediated collagen expression

**Figure 3:**
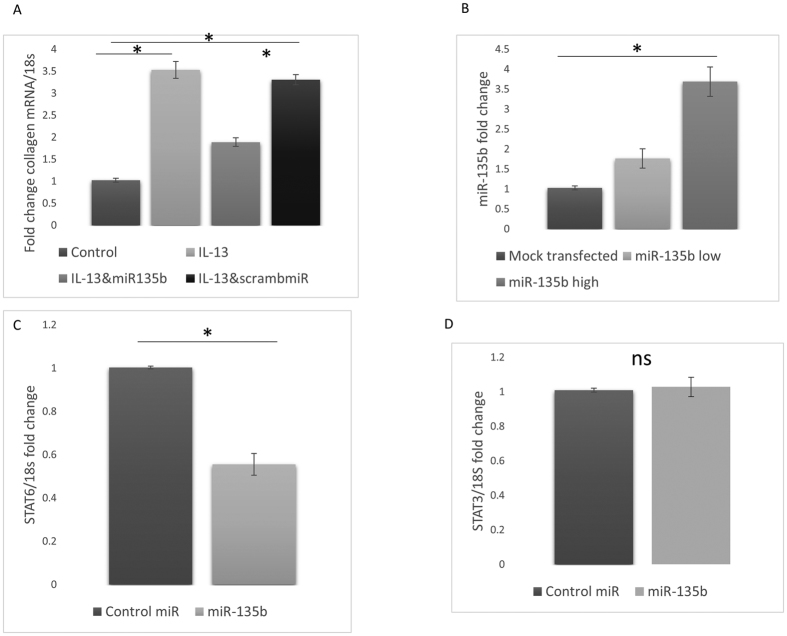
miR-135b targets STAT6 to reduce collagen induction. (**A**) Dermal fibroblasts were transfected with miR135b mimics or concentration matched control miR (*C. elegans*) for 24 hours after which time the medium was replenished and contained 100 ng/ml recombinant IL-13 for a further 24 hours after which time the cells were harvested and qPCR was performed for collagen1A1 and normalised to 18 s. Data is the mean and SD n = 5 five different donors. (**B**) 75 nM of 135b mimic (low dose) or 150 nM (high dose) was transfected into dermal fibroblasts and after 24 hours post transfection miR135b was quantitated by qRT-PCR, data is the mean and standard deviation. (**C**) 75 nM of 135b mimic was transfected into dermal fibroblast and after 24 hours post transfection STAT6 was quantified by qRT-PCR, data is normalised to 18 S and compared to scrambled miR transfected n = 5 from five different individual donors. (**D**) STAT3 levels were analysed by qPCR after transfection with miR135b mimic or control miR 24 hours post transfection. Data is the mean and SD. ns: no significant difference between groups.

We then used computational algorithm prediction software to identify possible miRs that target the downstream signal molecule STAT6. These computer prediction software work on the basis of putative seed pairing between the ‘seed’ region in the miR and the 3′UTR in its target mRNA thus leading to target mRNA repression. These identified miR-135b as a miR likely to target STAT6 directly through this seed region. Transfection of synthetic miR-135b mimics into dermal fibroblasts attenuated the IL-13 induction of collagen1A1 expression; however the scrambled miR did not attenuate the collagen induction by IL-13 stimulation (100 ng/ml) Fig. 3A (Significantly different between control vs IL-13 treated and IL-13 treated plus miR135b mimic. No significant difference between IL-13 treated vs IL-13 treated and scrambled miR mimic, ANOVA). To confirm that transfection of miR-135b into the fibroblast was efficient in these cells we transfected a high and low concentration and then measured the miR levels 24 hours later using qRT-PCR. Figure 3B demonstrates the expression of miR-135b after transfection of low and high dose miR-135b. Figure 3C also demonstrates that transfection of miR-135b alone reduces STAT6 mRNA levels significantly. However, it did not alter the levels of a similar STAT, STAT3 (Fig. 3D). Interestingly, miR-135b has a binding site for the 3′UTR of Monocyte Chemoattraction Protein 3/CCL7 and should target this directly we therefore measured MPC-3 after transfection of miR-135b. There was a reduction after transfection of miR-135b of MCP-3 gene expression.

### Reduced levels of miR-135b in SSc fibroblasts and elevated STAT6

**Figure 4:**
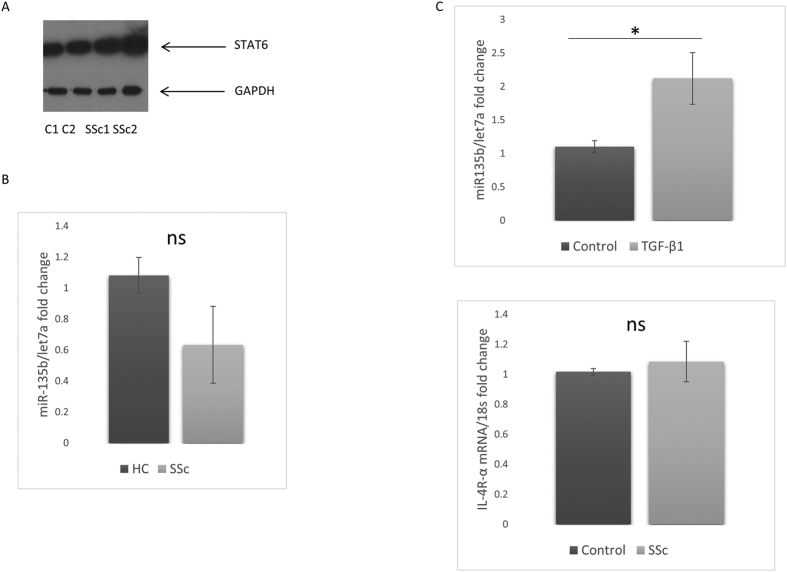
Elevated miR135b target STAT6 SSc fibroblasts. (**A**) Dermal fibroblasts or SSc fibroblasts were isolated and cultured within the first three passages the cells were lysed and a western blot was performed for STAT6. Representative western blot from 2 individual donors. GAPDH was used as the loading control for equal loading. (**B**) Healthy and SSc fibroblasts the RNA was harvested and qRT-PCR was performed for the expression of miR-135b and normalised to the expression of let7a. Data is the mean and SD n = 3. No significant difference. (**C**) Down regulation of miR-135b by TGF-β1. Dermal fibroblasts were stimulated with TGF-β1 (10 ng/ml) and after 24 hours the RNA was harvested and qRT-PCR was performed for miR-135b and normalised to the expression of let7a. Data is the mean and SD n = 8. Significantly different compared to control (Student’s t test). (**D**) SSc dermal fibroblasts express IL-4 Rα chain. Fibroblasts were isolated and cultured and then RNA harvested then qRT-PCR was performed for IL-4 Rα and normalised to 18S. Data is the mean and SD n = 3. ns: no-significant difference between groups.

The target of miR135b is STAT6 due to the prediction software analysis and also because of the reduction of STAT6 with synthetic miRs we measured the levels in dermal fibroblasts in SSc patients and found this to be elevated (Fig. 4A). Because we had found that miR-135b was regulating STAT6 and mediating collagen expression we examined the levels of miR-135b in SSc dermal fibroblasts. We found a reduced expression of miR-135b levels in SSc compared to control fibroblasts, however, this did not quite reach statistical significance (Fig. 4B). Because TGF-β1 is a critical cytokine in fibrosis and is highly elevated in SSc we treated cells with TGF-β1 (10 ng/ml) to see if this reduced miR-135b. Unexpectedly, TGF-β1 stimulation led to a 2 fold increase miR-135b expression compared to untreated healthy control fibroblasts (*P* = 0.0354 Student’s t-test) (Fig. 4C), this was opposite to what we had hypothesised.

Dermal fibroblasts isolated from SSc patients were examined for IL-4 Rα expression as this is the receptor subunit used by IL-13 (and IL-4) upstream of STAT6 and is common receptor through which both cytokines signal. There was no clear difference in expression of the IL-4 receptor in SSc fibroblasts (Fig. 4D). Because reactive oxidative species has been shown to play a prominent role in fibrosis in SSc we incubated healthy control dermal fibroblast cells with sub toxic concentrations H202 to examine its effect on IL-4Rα expression. MTT assay demonstrated that the H202 treated cells were not dying. qPCR demonstrated no significant difference in IL-4R expression between H202 treated and non-treated cells (*P* => 0.05; Student’s t test).

### Levels of serum and monocyte miR-135b

**Figure 5:**
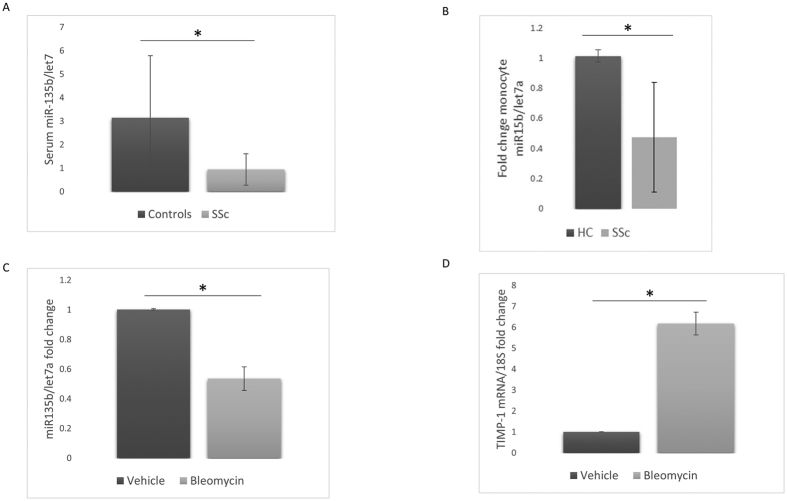
SSc sera has reduced miR-135b levels. (**A**) SSc and healthy control serum was measured for miR-135b and let7a by qRT-PCR. Data is the mean and SD of HC = 12 and SSc = 15 (*P* = 0.006 Student’s t-test). (**B**) CD14+ cells were isolated and levels of miR-135b and let7a were measured by qRT-PCR. Data is the mean an SD n = 7 (*P* = 0.03 Student’s t-test). (**C**) Bleomycin treated fibrotic mice have diminished miR-135b levels. C56/B Mice were vehicle or bleomycin treated and after fibrosis established skin biopsies were taken and the levels of miR135b were measured by RT qPCR and the data was normalised to let7a. Data is the mean and SD of five animals per group n = 5 (*P* = 0.0001 Student’s t test). (**D**) TIMP-1 levels were analysed by qPCR. Data is the mean and SD of five animals per group n = 5 (*P* = 0.0001 Student’s t test).

It has recently been discovered that miRs can also be extracellular and that these miRs are enclosed within vesicles called exosomes rendering them remarkably stable. Patient and healthy control serum was isolated and measured for miR-135b by qRT-PCR and this demonstrated highly significantly reduced levels of miR-135b compared to healthy controls (*P* = 0.0060 Student’s t test) (Fig. 5A). To elucidate this further we measured the levels of miR-135b in isolated CD14+monocytes in patients and found this was significantly lower compared to controls (Fig. 5B).

### Bleomycin treated mice have lower miR-135b levels

A useful model of skin fibrosis is the bleomycin model in which instillation of the compound bleomycin into the mouse results in inflammation-driven fibrosis that recapitulates the fibrosis in SSc. We measured the levels of miR-135b in the skin of mice treated with bleomycin or vehicle control treated mice and found highly significantly reduced levels of miR-135b (*P* = 0.0001; Student’s t test) Fig. 5C). Pro- fibrotic TIMP-1 mRNA was also significantly elevated in the bleomycin mouse tissue compared to vehicle control (Fig. 5D).

### Regulation of miR135b levels

**Figure 6:**
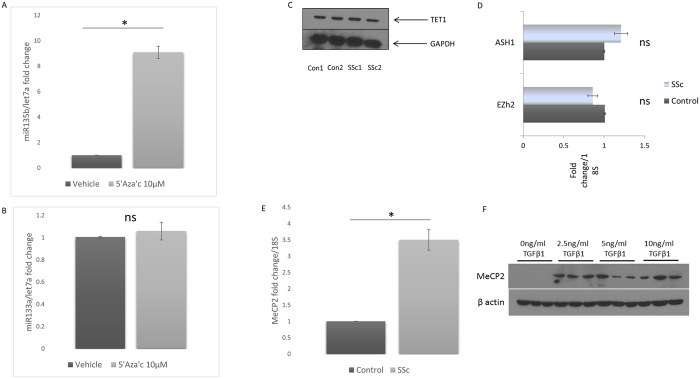
Methylation regulates miR135b. (**A**) Healthy dermal fibroblasts were treated with vehicle control (DMSO) or 10 μM 5′Aza′c for 5 days in culture after which qPCR was performed for miR135 and let7a data is normalised to let7a and compared to vehicle control data is the mean and SD of 5 individual donors n = 5. (**B**) MiR133a levels were measured by qPCR and normalised to let7a. Data is the mean and SD of five individual donors n = 5. (**C**) Healthy and SSc dermal fibroblasts express similar levels of the DNA modifying enzyme TET1. Representative western blot for TET1 from two individual donors with limited SSc. GAPDH is used as the loading control n = 4. (**D**) Healthy or SSc dermal fibroblasts were analysed by qPCR for the genes Ezh2 and ASH1 and data normalised to 18 s. The data is the mean and SD n = 4 ns: no significant difference between groups. (**E**) SSc fibroblasts express higher MeCP2 levels. Heathy or SSc dermal fibroblasts were analysed by qPCR for MeCP2 expression and the data normalised to 18 S. The data is the mean and SD n = 4 (*P* = <0.001 Student’s t test). (**F**) Western blot of fibroblasts treated with increasing doses of TGF-β1 for MeCP2 levels. Β actin served as a loading control.

Because the miR appears to be regulating its target STAT6 and we had demonstrated STAT6 is altering collagen levels and in SSc cells the miR was downregulated we wondered what is regulating the repression of the miR itself? Examining the promoter sequence of the miR135b gene it could be seen that it has CpG sites that could be methylated we therefore incubated healthy dermal fibroblasts with the global demethylating agent 5′aza′C which sequesters DNMT enzyme activity. Incubation with 5′aza lead to significant upregulation of miR135b levels of 9 fold compared to vehicle treated cells (Fig. 6A) (P =<0.001 Student’s t test). However the same incubation of 5′azaC did not lead to upregulation of the unrelated microRNA miR133a compared to vehicle control treated cells (Fig. 6B). This indicates methylation is important in its regulation. In general hypermethylation leads to gene repression and hypomethylation leads to enhanced gene expression. Cytosine is the base that is methylated generating 5′methyl cytosine and it is now known that TET enzymes are the enzymes responsible for removing this modification and appear important in demethylation. We hypothesised that a reduction in the SSc dermal fibroblasts would be present leading to altered demethylation. Figure 6C demonstrated no difference in TET1 levels suggesting this is not a factor influencing this. We further examined the expression of the methyltransferase enzyme Enhancer of Zeste Homolouge 2 (Ezh2) which is important for the transfer of methyl groups and this was reduced in the SSc fibroblasts but not statistically significant (Fig. 6D). There was also no significant difference in the methyltransferase ASH1 (Fig. 6D). ASH1 is part of the trithorax proteins and was recently shown to regulate fibrosis by binding directly to the TIMP-1 promoter.

Methyl cap binding protein 2 (MeCP2) is the protein most associated with the disease Rett syndrome but is ubiquitously expressed^21^. This acts by binding to DNA to repress the DNA gene expression by binding in its methyl binding domain. Because of its repressive function in DNA we examined its expression. We found that MeCP2 is significantly enhanced in SSc dermal fibroblasts compared to control fiboblastss (Fig. 6E). Furthermore in normal dermal fibroblasts exposure to TGF-β1 leads to significant elevation of MeCP2 expression, but does not appear to be dose dependant (Fig. 6F).

### Demethylation reduces collagen levels

**Figure 7:**
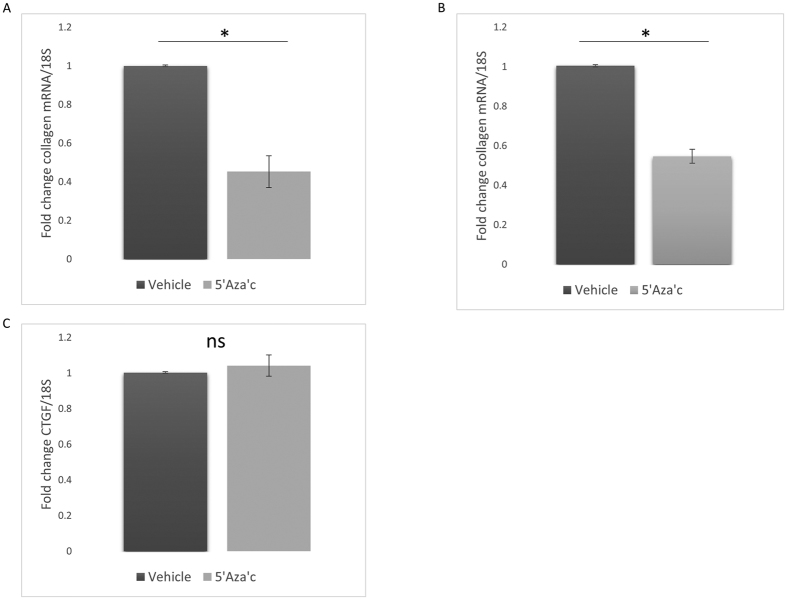
Treatment with the global hypomethylating compound 5′Aza′c reduced collagen levels. (**A**) Healthy dermal fibroblasts were treated with DMSO vehicle control or 5′Aza′c for four days after which qPCR was performed for collagen1A1 and 18 S. Data is the mean and SD of five donors and was normalised to 18S n = 5. (**B**) SSc dermal fibroblasts were treated with DMSO vehicle control or 5′Aza′c for four days after which qPCR was performed for collagen and 18S. Data is the mean and SD normalised to 18S n = 4. (**C**) SSc dermal fibroblasts were treated with DMSO control or 5′Aza′c for four days after which CTGF and 18 S was measured by qPCR. Data is the mean and SD n = 4 ns: no significant difference between groups (Student’s t test).

We have shown that miR135b is regulated by methylation and that this could be mediated by the repressive protein MeCP2 so we examined the effects of 5′aza′c on collagen expression in fibroblasts. Exposure to 5′aza′C reduced the levels of collagen1A1 compared to vehicle control treated cells in healthy dermal fibroblasts (Fig. 7A). (P =<0.001 Student’s t test).

Using SSc dermal fibroblasts we also found a significant reduction in collagen levels after 5′azaC treatment also (Fig. 7B) (Student’s t test *P* =<0.05). However, we could see no significant difference in CTGF levels (Fig. 7C).

## Discussion

Extracellular Matrix (ECM) production involves responses to endogenous and exogenous factors that promote production of ECM or inhibit the breakdown of ECM. Excessive production leads to scarring and fibrosis and is especially prominent in SSc. IL-13 is a pro fibrotic molecule that is produced by T cells that activates ECM production and is a feature of many fibrotic diseases but its molecular mechanism is not well described. IL-13 is elevated in SSc and is associated with disease specific features such as nailfold capillary defects^5^ and IL-13 producing T cells are found within the skin. Here we demonstrate that it is STAT6-dependant and that miR-135b regulates STAT6 by targeting mRNA and that this miR may be reduced through a methylation dependant mechanism that may include MeCP2.

IL-13 has been demonstrated to be elevated in the bleomycin model of fibrosis and activation of the pregnane X receptor blocked the bleomycin induced fibrosis and this was not mediated directly but through reducing the expulsion of IL-13 from murine T cells to activate the fibroblasts to secrete ECM^22^, demonstrating the critical role of T cell mediated IL-13 in fibrosis. IL-13 has also been described to supress MMP-13 in isolated dermal fibroblasts thus decreasing the proteolytic degradation of ECM favouring an increase in deposition^23^ and also reduce MMP-3 release by ocular conjunctiva fibroblasts and in lung fibroblasts IL-13 has been shown to induce Platelet Derived Growth Factor (PDGF) release^24^.

We have previously demonstrated that T cell-derived IL-13 can induce collagen transcription in healthy dermal fibroblasts in culture^6^ and it is generally accepted that SSc has a Th2 dominance with increased Th2 cytokines both locally and in the sera^4^ and these foster the release of ECM. Furthermore, Fuschiotti *et al*. using multi colour flow cytometry demonstrated that it is effector CD8+T cells that are the highest producers of IL-13 in SSc, even though CD4+ T cells also display production^25^. They also found this CD8+ T cell production of IL-13 is associated with diffuse cutaneous SSc more so than limited SSc^25^. This has also been verified in a recent study with isolated CD8+ T cells^26^. It has also been shown that CD8+ T cells isolated from patients with SSc mediates increased collagen1A and fibronectin expression in dermal fibroblasts in culture and that this can be blocked by incubation with an anti-IL-13 antibody, but not by an anti-IL-4 antibody or isotype control indicating this is IL-13 specific and not mediated through IL-4. The authors also show that incubation of T cell supernatants with dermal fibroblasts promotes the phosphorylation of the transcription factor STAT6 assessed by flow cytometry. We had also seen a similar effect^6^ *in vitro*. Indeed siRNA mediated silencing of GATA3 in SSc CD8+ T cells from patients diminished high basal unstimulated levels of IL-13. It has been published that IL-13-mediated increases in lung fibrosis by a transgene expressing mouse was facilitated by TGF-β1 and can be reduced by blocking the activation of TGF-β^17^, however we did not find any increase in TGF-β levels after IL-13 stimulation nor the TGF-β activation protein TSP-1^19^. Furthermore blockade of the TGF-βR with chemical inhibition had no effect on collagen induction by IL-13. We also found no change in BAMBI levels which is a negative regulator of TGF-β signalling by acting as a pseudo receptor. This is in line with data in which IL-13 KO mice are protected from lung fibrosis but not IL-4 KO mice, despite the fact that they have abundant TGF-β1 levels. We demonstrate here that siRNA against STAT6 to silence the transcription factor diminishes the collagen induction mediated by IL-13 stimulation and this was also the case using the chemical STAT6 inhibitor that has previously been reported to inhibit STAT6 effects^20^. Thus, STAT6 is critical in mediating this response. However, it appears that TGF-β does not play a role in this induction. IL-13 had been previously shown in lung fibroblasts to induce collagen through TGF-β in fibroblasts from asthma patients^27^. It has recently been demonstrated that methylation of STAT6 determines its phosphorylation and modulates its DNA binding activity in response to exogenous stimulation^28^, thus adding another layer of complex regulation Because IL-13 shares its signalling with the shared co receptor IL-4α we measured the levels of this, we could find no increase in IL-4α in SSc fibroblasts compared to controls and this could not be modulated by oxidative stress either, as ROS is important in fibrogenesis.

Through computer prediction software we identified miR-135b as regulating STAT6 by binding to its 3′UTR region. We confirmed this with transfection of miR-135b and the reduction of STAT6 and augmentation of IL-13 induction of collagen expression in these cells. Therefore, reduction of miR-135b in SSc cells would lead to enhanced STAT6-mediated ECM induction. Interestingly we also observed reduction of MCP-3 which is predicted to be targeted by miR-135b as it has a binding site. This confirms transfection of the miR. MCP-3 is a chemoattractant for monocytes and monocytes are important in the disease and are often found in the skin of SSc patients. A study has shown that MCP-3 is elevated in SSc and the Tsk mouse model of fibrosis which has similar features to SSc. What actually modulates the levels of miR-135b is unknown and maybe a variety of soluble factors, possibly released by innate immune cells. We hypothesised that the pro fibrotic TGF-β1 would diminish miR-135b, however, it actually increased its expression. This may be part of a negative feedback loop to dampen down fibrosis, however further investigations are required.

Using the bleomycin model which recapitulates features of the disease, we found also that miR135b was significantly reduced in bleomycin treated mice. Many miRs have now been identified in mediating a role in fibrosis, however only a very few have been described in SSc. We also show reduced levels of miR135b in SSc dermal fibroblasts. It is known that miR-29a is involved in SSc^15^ and we have also demonstrated reduced levels of miR-29a in SSc dermal fibroblasts^29^ and this targets collagen. Others have demonstrated down regulated miR let7-a in SSc by *in situ* hybridisation and qRT-PCR and found that this contributes to excessive collagen production^30^. Similarly in keloid fibroblasts, which are abnormally excessive ECM type fibroblasts aberrant expression of miRs has been demonstrated to mediate collagen1 expression^31^. The finding that miR-135b regulates collagen expression via direct targeting of STAT6 further supports the notion that dysregulated miRs play a role in fibrosis.

MiRs are very stable in serum and are released enclosed within membrane bound vesicles called exosomes. These exosomes appear to protect the miR from endogenous RNAses that would otherwise degrade the miR. We demonstrate for the first time reduced serum miR135b levels in SSc patients compared to healthy controls. The significance of this is unknown but in further larger studies if this is replicated this may be a potential biomarker. Because there were differences in serum levels we sought to identify another cell type that might responsible for this, We found significantly reduced levels in CD14+ monocytes in SSc patients. The significance of this is not clear but one would expect its target STAT6 also to be elevated in the monocytes. STAT6 in monocytes is important in M2 differentiation and these are important in wound healing.

We sought to identify what is underlying the repression of miR135b and because it was noted that it has CpG sites that can be methylated in its promoter we hypothesised that the miR is hypermethylated. We show through the use of the hypomethylating agent 5′aza′C that the miR was elevated after treatment compared to vehicle control and furthermore an unrelated miR, miR133a was not changed, suggesting that this is a specific event rather than a global one. Because it is now known that TET1 enzyme is critical in the demethylation we examined the expression of this enzyme in healthy and SSc fibroblasts and found no significant difference between the groups.

MeCP2 is a methylated binding protein that binds to methylated DNA to facilitate a repressive state^21^ and we hypothesised this may be modulated in SSc. We examined the levels of MeCP2 and found this to be elevated in SSc fibroblasts. MeCP2 is part of a family of methyl CpG binding proteins and is the most abundantly expressed member of this family^21^. Mutations in the protein are more commonly associated with Rett syndrome a rare X-linked neurodevelopmental disorder affecting 1 in 10000 females^32^. Mutations are often found in the methyl binding domain of the protein and lead to alterations in its levels in the brain, however, it precise molecular effects are unknown. We demonstrate that elevated levels of MeCP2 may be associated with repression of miR135b and that this could be forming a complex with HDAC1. We also found large upregulation of MeCP2 in healthy cells upon TGF-β1 stimulation. It is of note that a SNP in MeCP2 is associated with SSc^33^. Furthermore, MeCP2 heterozygous mice have attenuated fibrosis in the liver after carbon tetrachloride instillation with much lower myofibroblasts and collagen content^34^. Furthermore MeCP2 has been found in fibrotic hearts^35^. This indicates that MeCP2 is critical in fibrosis. Interestingly their also appears to be some interaction in liver fibrosis between MeCP2 and the methyltransferase ASH1, where the increased expression of MeCP2 either through regulation of miRs or another mechanism appears to elevate ASH1 levels and increases ECM through direct binding of the genes in their promoters. Although we saw some elevation in SSc dermal fibroblasts of ASH1 this was not statistically significant. It is suggested that the repression of miR135b is due to methylation as there is methylation sites in the CpG islands in the miR135b promoter and due to the fact that hypomethylation led to an increase in miR levels. Although 5′Aza′c is none specific in demethylating it is suggested that it is demethylating the miR135b promoter and thus derepressing this. This repression may be aided by MeCP2 that is binding the methylated DNA and helping to repress it possibly in combination with HDAC1. In liver fibrosis this repression by MeCP2 and HDAC1 leads to repression of PPAR-γ and consequent silencing of this gene, PPARγ itself is a negative regulator of fibrosis and the reduction of this releases the ECM genes to be expressed^34^. Also a transgenic mouse over expressing MeCP2 has exacerbated heart fibrosis and enhanced scaring in response to pressure overload^36^.

Finally as well as the DNA demethylation increasing the levels of the miR it also demonstrated a reduction in collagen expression.

In SSc dermal fibroblasts it has been shown that 5′Aza′C reduced hypermethylation of the Wnt antagonist DKK1 and Secreted Frizzled RP1 (SFRP1) and thus enhanced their expression and this was associated with a blockade of pro-fibrotic Wnt signalling and *in vivo* 5′Aza′C ameliorated bleomycin induced fibrosis^37^. The blockade of fibrosis in the bleomycin model by hypomethylation could also have been due in part to reactivation of repressed miR135b. The fact that SSc fibroblasts in culture retain a fibrotic phenotype even over many passages suggests they have some sort of intrinsic ‘memory’, this could be due to the methylation that is imprinted and retained over multiple cell divisions. It has been demonstrated in cardiac fibroblasts that hypoxia induced fibrosis is driven by a global hypermethylation^38^. This increased methylation is genome-wide and mediated by a hypoxia inducible factor-dependant increase in DNMT3 as deletion *in vitro* of DNMT3 by siRNA reduced methylation and also collagen and α-Sma content^38^. This indicates the global alteration of methylation is important in the differentiation of fibroblasts to myofibroblasts. The initial event that is driving the methylation in their study is hypoxia and the heart is particularly vulnerable to a hypoxic state. In our study we do not know what is increasing methylation but it is interesting to note that hypoxia is prevalent in SSc and hypoxia has been shown to increase collagen expression in dermal fibroblasts^39^. It is suggested that hypoxia maybe leading to hypermethylation and increased ECM by repression of miR135b. It is also interesting to note that mice exposed to hypoxia experimentally have elevated ECM in their dermis^39^.

Immune perturbations are common in SSc and precede the fibrosis. Here we demonstrate that the T cell cytokine IL-13 mediates its effects through STAT6 and that STAT6-miR-135b are important in collagen increases. Reintroduction of miR-135b may be useful in STAT6-driven fibrosis. We also demonstrate the novel observation of elevated MeCP2 and that this may be important in SSc.

## Methods

This study was approved by the North East research ethics committee (REC no. 13/NE/0089 and 091/H0905/11) and all patients gave fully informed written consent. All methods were carried out in accordance with the approved guidelines. Normal dermal fibroblasts were isolated from healthy volunteers by skin punch biopsy (6 mm^3^) and from clinically diagnosed limited SSc patients from the affected skin area and the tissue was placed into a six well plate with Dulbeccos modified Eagles Medium (DMEM) medium (Sigma) supplemented with 10% (vol/vol) heat-inactivated Fetal Calf Serum (FCS) (Gibco, UK) and 2 mM L-glutamine and 100U/ml penicillin/streptomycin at 37 °C in an atmosphere containing 5% CO_2_. After a few days cells were confluent and passaged using trypsin/EDTA (Gibco, UK) from the explant and all experiments were performed in early passaged cells (p2–8). Control tissue was derived from healthy donors undergoing corrective abdominal reduction (plastic) surgery N = 5. SSc patients were all female and not taking any immunosuppressive therapy or chemotherapy and all had clinically diagnosed limited SSc n = 5 mean age 57 years old (all female SSc) taking no medication. The STAT6 inhibitor AS1517499^20^ was purchased from AxonMedchem (USA) and was used at a final concentration of 40 nM in cell culture experiments. The TGF-β inhibitor SB431542 (Tocris Biosciences, UK) was used at a final concentration of 10 μM to inhibit the TGF-β signalling system. Fibroblasts were pre-treated for 2 hours with or without SB431542 after which time IL-13 was incubated 100 ng/ml with the TGF-β inhibitor still within the media, 24 hours later the cells were harvested.

### RT-PCR

After cell stimulation cells were harvested in Trizol buffer and cellular RNA isolated according to the manufacturer’s instructions. RNA quality was assessed by nanodrop ND100 spectrophotometer with 260/280 nm readings. 1 μg RNA was reverse transcribed to cDNA with superscript III (Invitrogen, UK) with RNAse inhibitor. Prepared cDNA was subjected to quantitative PCR using SYBR green Taq ready (Sigma, UK) technology using specific primers to the genes of interest using the specific primers (forward and reverse) below in a total volume of 25 μl using the 7500 RT-PCR machine (Applied Biosciences, UK). Data was normalised to endogenous housekeeping gene 18 S, which was confirmed to be stable across the groups and relative changes were calculated using the deltadelta Ct method with the control as the comparator. Collagen1A1: forward 5′-CAAGAGGAAGGCCAAGTCGAGG-3′, reverse 5′-CGTTGTCGCAGACGCAGAT-3′ 18S Forward: 5′ CGAATGGCTCATTAAATCAGTTATGG-3′ Reverse 5′-TATTAGCTCTAGAATTAC CACAGTTATCC-3′. STAT6: Forward: 5′-CCTCGTCACCAGTTGCTT-3′ Reverse: 5′-TCCAGTGCTTTCTGCTCC-3′. STAT3: Forward: 5′-GGAGGAGTTGCAGCAAAAAG-3′ Reverse: 5′-TGTGTTTGTGCCCAGAATGT-3′.

Absent, Small or Homeotic disc-1 (ASH1): Forward: 5′AATGATCTTTGCTGAGTGTT-3′ Reverse: TCCCCAACCTTTTTCCTCAG-3′. Connective Tissue Growth Factor Forward: 5′-CTCGCGGCTTACCGACTG-3′ Reverse: 5′-GCACTTGAACTCCACCGG-3′ BMP And Activin Membrane-Bound Inhibitor (BAMBI): Forward: 5′-CGCCACTCCAGCTACATCTT-3′ Reverse: 5′-CAGATGTCTGTCGTGCTTGC-3′.

Alpha Smooth muscle actin: Forward: 5′-TGAAGAGCATCCCACCCT-3′ Reverse: 50-ACGAAGGAATAGCCACGC-3′. MCP-3/CCL7 Forward: 5′-TGTCCTTTCTCAGAGTGGTTCT-3′ Reverse: 5′-TGCTTCCATAGGGACATCATA-3′ IL-4 R Forward: 5′-CTGGAGCACAACATGAAAAGG-3′ Reverse: 5′-AGTCAGGTTGTCTGGACTCTG-3′. MeCP2: Forward: 5′-GATCAATCCCCAGGGAAAAGC-3′ Reverse: 5′-CCTCTCCCAGTTACCGTGAAG-3′.

Semi quantitative PCR for TGF-β1 was performed using primers for TGF-β1 Forward: 5′-GGATACCAACTATTGCTTCAGCTCC-3′ Reverse: 5′-AGGCTCCAAATATAGGGGCAGGGTC-3′ and the product ran on a 2% agarose gel containing ethidium bromide and visualised under UV light.

### ELISA for Thrombospondin-1 (TSP-1) and TGF-β1

Healthy dermal fibroblasts were seeded into 24-well plates and at 80% confluence serum starved for 24 hours after which recombinant IL-13 100 ng/ml or 10 ng/ml Transforming Growth Factor-β (a positive control) was added to serum free media and incubated for a further 24 hours. Conditioned medium was then collected and measured for TSP-1 using a human TSP-1 ELISA (R&D systems, UK) in accordance with the manufacturer’s instructions against a standard curve of recombinant TSP-1 using a fluorescent plate reader (Tecan). The same supernatants were used to measure TGF-β1 levels by ELISA (R&D systems, UK).

### siRNA knockdown

For the siRNA silencing experiments cells were seeded into 24-well plates, and at 50–60% confluence 100 nM STAT6 siRNA SMARTpool (Dharmacon, UK) 100 nM of none targeting control siRNA was transfected using DharmaFECT 1 in antibiotic and serum-free DMEM media. Following 24 h of incubation, medium was replaced with fresh medium FCS free medium supplemented with recombinant endotoxin-free IL-13 100 ng/ml (R&D systems, Abingdon, UK) and after a further 24 h cells were harvested for expression.

### Western Blotting

After transfection with specific siRNA or none targeting control siRNA (100 nM) cells were lysed in RIPA buffer containing protease inhibitors. 25 μg of total protein was loaded in Lammeli buffer with beta-mecaptoethanol and ran on a 10% Polyacrylamide denaturing gel. The gel was transferred to onto nitrocellulose membrane with wet transfer, blocked for 1 hour in 5% blocking solution (non-fat milk) in TBS-tween and then hybridised overnight with anti-STAT6 antibody (Cell signal, 9362) overnight 1:800 at 4 °C, washed three times in TBS-tween and then incubated with anti-Rabbit Horse Radish Peroxidase (HRP) antibody 1:4000 (Dako, UK) for 1 hour at room temperature, washed and then incubated with ECL substrate (Biorad, UK) and then exposed and developed. Healthy or SSc dermal fibroblast early passage were seeded and then lysed and then probed with anti-STAT6 1:800 or Ten Eleven Translocation-1 (TET1) (Abcam, UK) 1:700 or GAPDH (Abcam, UK) 1:8000. In some experiments healthy dermal fibroblasts where treated with increasing doses of recombinant TGF-β1 and then lysed and then probed for MeCP2 using an MeCP2 specific antibody (Ab2828) and the reprobed for β-actin (Ab8226).

### miR Experiments

miR-135b was identified by prediction software to bind the 3′UTR of STAT6 (Pictar software and Targetscan). Pre miR-135b (Thermo scientific, UK) was transfected into dermal fibroblasts at 75 nM concentration using dharmaFECT1 transfection reagent in antibiotic and FCS-free medium along with control miR (75 nM). The sequence of the control miR is designed to be complementary to the nematode worm *C. elegans*. To confirm successful transfection the miR was measured in the cells after transfection after three washes with sterile PBS (Sigma, UK) using qRT-PCR with specific primers for miR-135b (Applied Biosciences).

### H202 treatment

Healthy dermal fibroblasts were grown to confluence and then treated with H202 (Sigma, UK) at sub toxic concentrations 200 μM for 2 hours in FCS-free medium after 2 hours the H202 FCS-free medium was removed and replaced with complete medium containing 10% FCS and L-glutamine. These cells were left for 3 days after which RNA was isolated and cDNA generated and qPCR was performed for IL-4 Rα with the primers described in the section above.

### Measurement of miR-135b

MiR-135b was measured in SSc dermal fibroblasts and healthy control cells using qRT-PCR and normalised to let-7a levels using Taqman qPCR with primer specific reverse transcription. miR133a was also measured after 5′Aza′c treatment also. Serum levels of miR-135 was measured by qRT-PCR and also let7a after isolation with the miRNeasy RNA isolation kit (Qiagen, UK) following the manufacturer’s instructions. This serum miR study included larger number of patients as opposed to the dermal fibroblasts study. The number of patients was 15, n = 10 diffuse SSc and limited SSc patients n = 5. In some experiments healthy control dermal fibroblasts were treated with TGF-β1 (10 ng/ml) and after 24hrs RNA was harvested and qRT-PCR was performed for miR-135b and let7a as the endogenous internal control. In further experiments CD14+ monocytes where isolated from whole blood by negative selection and then RNA was isolated and miR-135b was measured along with let-7a.

### Collagen measurement

Total soluble collagen in cell culture supernatants was quantified using the SirCol collagen assay (Biocolor, Belfast, UK) as per the manufacturer’s instructions. Which is an assay based on hydroxyproline. The dermal fibroblast where treated with nothing or IL-13 (100 ng/ml) and after 24 hours the collagen protein was quantitated. Data was normalized to control which was set at 100% collagen.

### 5′aza′2-deoxycytidine( 5′aza′C) treatment

Human dermal fibroblasts were treated with 10 μM of 5′aza′C (Sigma, UK) or vehicle control DMSO for up to 5 days after which the miRs were measured as above. In separate experiments healthy and SSc fibroblasts were treated with 10 μM 5′aza′C or vehicle for 5 days and qPCR was performed for collagen, CTGF and 18 S.

### Bleomycin induced fibrosis

Wild type C57BL/6 mice were used in this study. All the mice where male and aged 8–10 weeks old at commencement of the study. Mice were anesthetised with isoflurane, their backs shaved and 100 μl 0.5 mg/ml bleomycin (Apollo Scientific, UK) or saline (vehicle control) administered via subcutaneous injection to an area approximately 1 cm^2^. Injections were repeated every other day for 4 weeks at which point mice were sacrificed (5 mice per group; treatment or vehicle).

### Wax embedded sample processing for miR quantification

Skin biopsies were taken from the vehicle treated and bleomycin treated mice placed in 10% formaldehyde for 16 hours after which time they were dehydrated through graded ethanols and then placed in zylene, samples were then embedded into wax and then sectioned. 10μm sections were then used to isolate RNA using the miRNeasy FFPE kit (Qiagen, UK) which includes removing from the paraffin wax and treating with proteinase K digestion and isolation according to the manufacturer’s protocol. Tissue isolated RNA was measured for quality and reverse transcribed for miR135b and let7a (Applied Biosystems; Id 4427975) using qRT-PCR with taqman™ probes. Data is normalised to let7a and shown as fold change compared to vehicle treated mice. Sections were also taken for the analysis of TIMP-1 by qPCR and normalised to 18 s as the internal housekeeper. Mouse TIMP-1 Primers: Forward: 5′-CAGTAAGGCCTGTAGCTGTGC-3′ and 5′-CTCGTTGATTTCGGGGAAC-3′.

### Statistical analysis

Statistical analysis was performed between groups with a Student’s T test or Analysis of Variance (*ANOVA)* with *P* values < 0.05 considered statistically significant * indicates significance in graphs.

## Additional Information

**How to cite this article**: O’Reilly, S. *et al*. IL-13 mediates collagen deposition via STAT6 and microRNA-135b: a role for epigenetics. *Sci. Rep.* **6**, 25066; doi: 10.1038/srep25066 (2016).
