# Corrigendum: IL-13 mediates collagen deposition via STAT6 and microRNA-135b: a role for epigenetics

**DOI:** 10.1038/srep27325

**Published:** 2016-06-10

**Authors:** Steven O’Reilly, Marzena Ciechomska, Nicola Fullard, Stefan Przyborski, Jacob M. van Laar

Scientific Reports 6: Article number: 25066; 10.1038/srep25066 published online: 04262016; updated: 06102016

This Article contains errors.

Marzena Ciechomska is incorrectly affiliated with ‘Institute of Cellular Medicine Faculty of Medical Sciences, MRG 4th Floor Cookson Building Framlington Place, Newcastle Upon Tyne, NE2 4HH’ and ‘Institute of Immunology and Experimental Therapy, Polish Academy of Science, Wroclaw, Poland’. The correct affiliation is listed below:

National Institute of geriatrics rheumatology and rehabilitation, Warsaw, Poland.

In the Author Contributions statement,

“S.O.R. performed experiments and wrote the manuscript. M.C. performed some experiments and helped draft the manuscript. N.F. and S.P. performed some experiments. J.M.v.L. drafted and commented on the manuscript and had oversight of the project”.

should read:

“S.O.R. performed experiments and wrote the manuscript. MC, NF and SP performed some experiments and helped draft the manuscript. J.M.v.L. drafted and commented on the manuscript and had oversight of the project”.

